# The Model for End-Stage Liver Disease (MELD) Score Predicting Mortality Due to SARS-CoV-2 in Mexican Patients

**DOI:** 10.3390/jcm13195777

**Published:** 2024-09-27

**Authors:** José Manuel Reyes-Ruiz, Ana Citlali Avelino-Santiago, Gustavo Martínez-Mier, Claudia Vanessa López-López, Luis Adrián De Jesús-González, Moises León-Juárez, Juan Fidel Osuna-Ramos, Carlos Noe Farfan-Morales, Selvin Noé Palacios-Rápalo, Víctor Bernal-Dolores, Rosa María Del Ángel

**Affiliations:** 1 Unidad Médica de Alta Especialidad, Hospital de Especialidades No. 14, Centro Médico Nacional “Adolfo Ruiz Cortines”, Instituto Mexicano del Seguro Social (IMSS), Veracruz 91897, Mexico; ani.avelino@hotmail.com (A.C.A.-S.); gustavo.martinezmi@imss.gob.mx (G.M.-M.); victor.bernal@imss.gob.mx (V.B.-D.); 2 Facultad de Medicina, Complejo Regional Sur Tehuacán, Benemérita Universidad Autónoma de Puebla (BUAP), Puebla 7585, Mexico; 202129601@viep.com.mx; 3 Programa Interinstitucional para el Fortalecimiento de la Investigación y el Posgrado del Pacífico (Programa Delfín), Tepic 63000, Mexico; 4 Unidad de Investigación Biomédica de Zacatecas, Instituto Mexicano del Seguro Social, Zacatecas 98000, Mexico; luis.dejesus@cinvestav.mx; 5 Laboratorio de Virología Perinatal y Diseño Molecular de Antígenos y Biomarcadores, Departamento de Inmunobioquimica, Instituto Nacional de Perinatología, Mexico City 11000, Mexico; moisesleoninper@gmail.com; 6 Facultad de Medicina, Universidad Autónoma de Sinaloa, Culiacán 80019, Mexico; osunajuanfidel.fm@uas.edu.mx; 7 Departamento de Ciencias Naturales, Universidad Autónoma Metropolitana (UAM), Unidad Cuajimalpa, Mexico City 05348, Mexico; 8 Department of Infectomics and Molecular Pathogenesis, Center for Research and Advanced Studies (CINVESTAV-IPN), Mexico City 07360, Mexico; rmangel@cinvestav.mx; 9 Institut Pasteur, Unité de Virologie Structurale, 75015 Paris, France; selvin.palacios@cinvestav.mx

**Keywords:** COVID-19, SARS-CoV-2, model for end-stage liver disease (MELD) score, biomarker, mortality

## Abstract

**Background/Objectives**: Coronavirus Disease 2019 (COVID-19) can cause liver injury and a deterioration of hepatic function. The Model for End-Stage Liver Disease (MELD) score is a good predictor for poor prognosis of hospitalized COVID-19 patients in the United States, Egypt and Turkey. Nevertheless, the best cut-off value for the MELD score to predict mortality in the Mexican population has yet to be established. **Methods**: A total of 234 patients with COVID-19 were studied in a tertiary-level hospital. Patients were stratified into survivors (*n* = 139) and non-survivors (*n* = 95). Receiver operating characteristic curves, Cox proportional hazard models, Kaplan–Meier method, and Bonferroni corrections were performed to identify the predictors of COVID-19 mortality. **Results**: MELD score had an area under the curve of 0.62 (95% CI: 0.56–0.68; *p* = 0.0009), sensitivity = 53.68%, and specificity = 73.38%. Univariate Cox proportional hazard regression analysis suggested that the leukocytes > 10.6, neutrophils > 8.42, neutrophil-to-lymphocyte ratio (NLR) > 8.69, systemic immune-inflammation index (SII) > 1809.21, MELD score > 9, and leukocyte glucose index (LGI) > 2.41 were predictors for mortality. However, the multivariate Cox proportional hazard model revealed that only the MELD score >9 (Hazard Ratio [HR] = 1.83; 95% confidence interval [CI]: 1.2–2.8; *P*_corrected_ = 0.03) was an independent predictor for mortality of COVID-19. **Conclusions**: Although the MELD score is used for liver transplantation, we suggest that a MELD score >9 could be an accurate predictor for COVID-19 mortality at admission to ICU requiring mechanical ventilation.

## 1. Introduction

Since 2020, the coronavirus disease 2019 (COVID-19) pandemic has been one of the main risks to public health worldwide as an outcome of severe acute respiratory syndrome coronavirus 2 (SARS-CoV-2) infection, belonging to the *Coronaviridae* [1]. COVID-19 causes a syndrome with non-specific characteristics that includes fever, dry cough, dyspnea, and fatigue, but it can also harm the organs, including the liver [2]. Alterations in liver function tests, mainly manifested by a significant elevation of hepatic enzymes and bilirubin in hospitalized patients with COVID-19 are evidence of the existing liver damage associated with SARS-CoV-2 infection [3,4]. COVID-19-associated liver injury has been defined as any hepatic damage that occurs during the patient’s illness and treatment with COVID-19, with or without a history of liver disease, causing an unfavorable prognosis and higher risk of mortality [4,5]. The damage caused to the liver during SARS-CoV-2 infection could be related to the direct cytopathic effect on hepatocytes and underlying organs or the hyperinflammation and cytokine storm [6].

The model for end-stage liver disease (MELD score), which is calculated with three standard laboratory variables (international normalized ratio (INR), serum creatinine, and total bilirubin), was created as a predictive value for mortality in patients with trans-jugular intrahepatic portosystemic shunts by complications of portal hypertension [7]. MELD score also has application in chronic liver disease and liver transplant allocation [7]. Studies that were performed in the African, Asian and North American populations have shown the usefulness of the MELD score for predicting the risk of hospital mortality in patients with COVID-19 [8,9,10]. However, more information is needed on the prediction capacity of this biomarker in other populations. Therefore, this paper aimed to assess the predictive value of the MELD score for mortality of patients with COVID-19 in the Mexican population without a history of chronic liver disease. For this study, the MELD score was compared with the biomarkers for SARS-CoV-2 infection to extend the knowledge available on the potential of the predictors of mortality at admission to ICU requiring mechanical ventilation.

## 2. Materials and Methods

### 2.1. Study Design and Participants

This retrospective and cross-sectional study was conducted following the Declaration of Helsinki at a tertiary care hospital from the Mexican Social Security Institute (IMSS). This study was approved in 2024 by the local research ethical Committee (R-2024-3001-113). Patients diagnosed with COVID-19 by a positive nucleic acid test for SARS-CoV-2 and admitted from June to December 2021 to the intensive care unit (ICU) with acute respiratory distress and invasive mechanical ventilation were screened for inclusion in the study. Subjects with the following characteristics were excluded from the study: aged less than 18 years, acquired immune deficiency syndrome, liver cirrhosis, liver cancer, malignant tumor, non-alcoholic and alcoholic fatty liver diseases, and pregnancy. All the patients were admitted for COVID-19 to the ICU, and the patients with other causes of mortality and additional SARS-CoV-2 infection were excluded from the study.

### 2.2. Data Collection

All laboratory (leukocytes, neutrophils, lymphocytes, platelets, glucose, urea, creatinine, total bilirubin, aspartate aminotransferase (AST), alanine aminotransferase (ALT)), and demographic (sex, age, height, weight, body mass index (BMI), diabetes, and hypertension) data were obtained from the electronic health records. Blood samples for hematologic and biochemical parameters assessment were collected within 24 h after admission and measurements within 1 h of sample collection. The routine blood tests were analyzed using the Sysmex XN-1000 Automated Hematology Analyzer (Sysmex, Kobe, Japan) and 3-level quality controls (Hematology Control XN Check). VITROS 4600 Chemistry System (Ortho Clinical Diagnostics, Raritan, NJ, USA) was used to assess the biochemical parameters.

### 2.3. Outcome and Exposure Variable

The in-hospital mortality of the COVID-19 hospitalized patients was evaluated as an outcome variable. The mortality of the patients after discharge from the hospital was not considered. MELD score was the exposure variable and was calculated using bilirubin, creatinine, and international normalized ratio (INR) with the formula 9.57 × loge (creatinine) + 3.78 × loge (total bilirubin) + 11.2 × loge (INR) + 6.43 [7]. The length of hospital stay was considered for the analysis of Cox and Kaplan–Meier.

### 2.4. Laboratory Biomarkers Reported for COVID-19

The AST-to-lymphocyte ratio index (ALRI) was provided by the formula ALRI = aspartate aminotransferase (U/L)/lymphocyte [11]. APRI was given by AST (U/L)/upper limit of normal value (U/L)/platelet count × 100 [12]. AST-to-neutrophil ratio index (ANRI) was obtained by dividing the AST (U/L) by the neutrophil count [13]. Neutrophil/lymphocyte ratio (NLR) = the total absolute neutrophil counts over total lymphocyte counts [14]. Platelets-to-lymphocyte ratio (PLR) = absolute platelet count/absolute lymphocyte count [15]. SII = platelet count × neutrophil count/lymphocyte count (×10^9^/L) [16]. The leukocyte glucose index (LGI) was defined as the product between blood leukocytes counts and glucose levels divided by 1000 [17]. Lactate dehydrogenase (LDH)/lymphocyte ratio = LDH levels (U/L)/lymphocyte counts (cells/µL) [18]. Blood urea nitrogen/creatinine (BUN/Cr) ratio = BUN/Cr (mg/dL) [19].

### 2.5. Statistical Analysis

Quantitative variables were expressed as mean ± standard deviation or median (interquartile range). Categorical variables were described as numbers (%). The distribution of quantitative variables was evaluated using the Shapiro–Wilk test, Q-Q plots, and histograms. Mann–Whitney test or Student’s *t*-test were used to compare continuous variables between the two cohorts. The Chi-square test was used for categorical variables. Spearman correlation coefficient was used to determine the correlations between continuous variables. The accuracy of the hematologic and biochemical parameters in predicting COVID-19 mortality was calculated using the Receiver Operator Characteristic (ROC) curve, obtaining the area under the curve (AUC), specificity, and sensitivity. Youden’s-J index was used to find the best cut-off value. Leukocytes, neutrophils, NLR, SII, MELD score and LGI levels were categorized into two groups by defining the best cut-off obtained of Youden’s-J statistic on the ROC curve. Survival curves were estimated using the Kaplan–Meier method, where the 22-day outcomes of these groups were assessed using the long-rank test. Univariate and multivariate Cox proportional hazards regression analysis were performed to calculate the hazard ratio (HR) with a corresponding 95% confidence interval (CI). The variables showing significant results in the univariate Cox regression analysis were selected to be tested in the multivariate Cox regression analysis. Univariate and multivariate logistic regression analysis was performed to determine the predictive factors for MELD score >9 in patients with COVID-19. The Odds Ratios (OR) with a corresponding 95% CI were calculated. A *p*-value < 0.05 was defined as statistically significant. *p* values were adjusted using the Bonferroni corrections (*P*_corrected_) to compensate for the effect of multiple hypothesis testing. All statistical analyses were performed with MedCalc v.18.2.18, SPSS Statistics v.26, and R v4.03 Statistical Software.

## 3. Results

### 3.1. Sample Overview

A total of 234 patients with COVID-19 were included in this study, among which were 139 surviving and 95 non-surviving. The mean age of non-survival was 65.23 ± 13.86, and the survival group was 62.74 ± 12.678 (*P*_corrected_ = 0.190). The median fraction of the surviving patients’ inspired oxygen (FiO_2_) and oxygen saturation (SpO_2_) were 41% and 71%, respectively, whereas the patients who did not survive showed a FiO_2_ and SpO_2_ of 60% and 58.5%, respectively. The leukocytes, neutrophils, NLR, SII, MELD, and LGI values were significantly older in the non-survivor group than in the survivor group (*P*_corrected_ < 0.05). The other biochemical and hematological parameters had no significant relationship between the groups studied (*P*_corrected_ > 0.05). The data for the patients are summarized in Table 1.

### 3.2. Assessment of the Hematologic and Biochemical Parameters for Predicting COVID-19 Mortality

**Figure 1:**
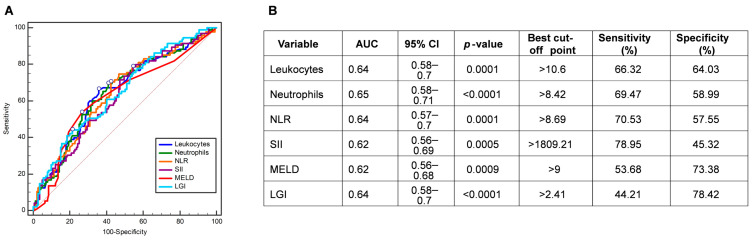
(**A**,**B**) Receiver operating characteristic (ROC) curves and accuracy evaluation of leukocytes, neutrophils, NLR, SII, MELD, and LGI in predicting COVID-19 mortality. Abbreviations: NLR, neutrophil-to-lymphocyte ratio; SII, systemic immune-inflammation index; MELD, Model of End-Stage Liver Disease Score; LGI, leukocyte glucose index.

ROC analysis in predicting COVID-19 mortality was performed for all variables that remained significant after the multiple hypothesis correction (*P*_corrected_ < 0.05) (Figure 1A). The AUC for leukocytes, neutrophils, NLR, SII, MELD, and LGI were 0.64 (sensitivity = 66.32%; specificity = 64.03%), 0.65 (sensitivity = 69.47%; specificity = 58.99%), 0.64 (sensitivity = 70.53%; specificity = 57.55%), 0.62 (sensitivity= 78.95%; specificity = 45.32%), 0.62 (sensitivity = 53.68%; specificity = 73.38%), and 0.64 (sensitivity = 44.21%; specificity = 78.42%), respectively (Figure 1B).

### 3.3. Survival Analysis Using Kaplan–Meier Curves

**Figure 2:**
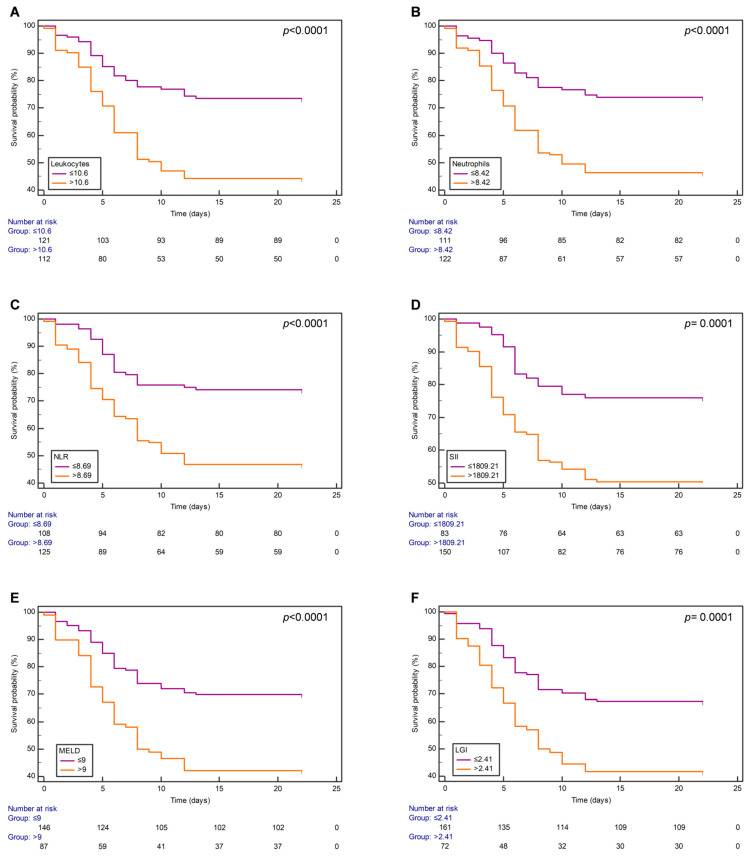
Kaplan–Meir survival curves of hospitalized COVID-19 patients according to established best cut-off values of (**A**) leukocytes, (**B**) neutrophils, (**C**) NLR, (**D**) SII, (**E**) MELD, and (**F**) LGI. Overall survival curves were estimated by Kaplan–Meier method and *p* values, by log-rank test. A *p* value < 0.05 was considered statistically significant. Abbreviations: NLR, neutrophil-to-lymphocyte ratio; SII, systemic immune-inflammation index; MELD, Model of End-Stage Liver Disease Score; LGI, leukocyte glucose index.

To identify the variables associated with COVID-19 mortality, the leukocytes, neutrophils, NLR, SII, MELD, and LGI were included in the analysis of Kaplan–Meier curves, which were created using the best cut-off points for each variable. COVID-19 patients above the stated leukocytes (>10.6 = 12.72 days vs. ≤10.6 = 17.68 days; *p* < 0.0001), neutrophils (>8.42 = 13.07 days vs. ≤8.42 = 17.74 days; *p* < 0.0001), NLR (>8.69 = 13.15 days vs. ≤8.69 = 17.77 days; *p* < 0.0001), SII (>1809.21 = 13.69 days vs. ≤1809.21 = 18.19 days; *p* = 0.0001), MELD score (>9 = 12.31 days vs. ≤9 = 17.08 days; *p* < 0.0001), and LGI (>2.41 = 12.16 days vs. ≤2.41 = 16.67 days; *p* = 0.0001) best cut-off values had a lower median survival time compared to those with values less than or equal to the best cut-off point (Figure 2A–F). All the parameters showed a significant difference (Log-rank (Mantel-Cox) < 0.0005) in survival between the between the high and low groups according to the best cut-off point at 22 days.

### 3.4. MELD Score Is a Predictor of COVID-19 Mortality

The univariate Cox regression model suggested that leukocytes >10.6 (HR = 2.53, 95% CI: 1.66–3.87; *P*_corrected_ = 0.0001), neutrophils >8.42 (HR = 2.45, 95% CI: 1.59–3.79; *P*_corrected_ = 0.0003), NLR > 8.69 (HR = 2.46, 95% CI: 1.58–3.82; *P*_corrected_ = 0.0003), SII > 1809.21 (HR = 2.49, 95% CI: 1.52–4; *P*_corrected_ = 0.001), MELD score > 9 (HR = 2.34, 95% CI: 1.56–3.5; *P*_corrected_ = 0.0001), and LGI > 2.41 (HR = 2.17, 95% CI: 1.45–3.25; *P*_corrected_ = 0.0006) were predictors of in-hospital mortality (Table 2). However, multivariate Cox regression revealed that only MELD score > 9 (HR = 1.83, 95% CI: 1.2–2.8; *P*_corrected_ = 0.030) was a prognostic factor for COVID-19 mortality (Table 2).

### 3.5. Correlation Analysis between MELD Score and Inflammation-Related Parameters in Patients with COVID-19

**Figure 3:**
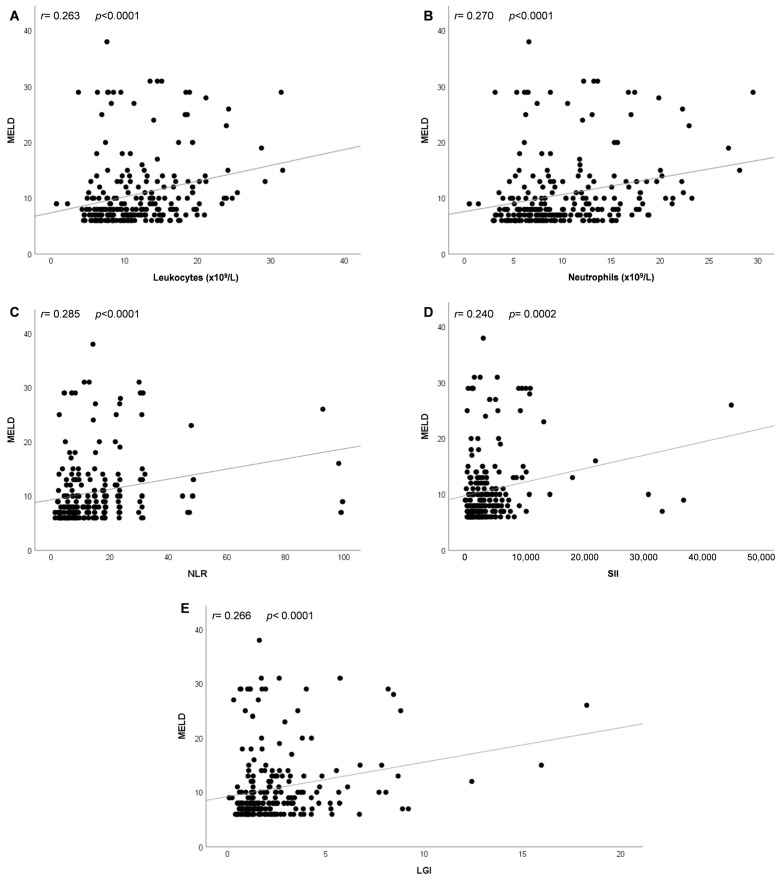
Spearman correlations between values of the MELD score and the inflammation-related parameters in patients with COVID-19. Scatter plots showing the correlation between MELD and (**A**) leukocytes, (**B**) neutrophils, (**C**) NLR, (**D**) SII, and (**E**) LGI. Spearman’s test was used to evaluate the correlations. A *p* value < 0.05 was considered statistically significant. Abbreviations: NLR, neutrophil-to-lymphocyte ratio; SII, systemic immune-inflammation index; MELD, Model of End-Stage Liver Disease Score; LGI, leukocyte glucose index.

The correlation between the MELD score and the inflammation-related parameters included in the Cox regression model was analyzed using Spearman’s correlation coefficient. MELD score had a weak positive correlation with leukocytes (*r* = 0.263 [95% CI: 0.139–0.378]; *p* < 0.0001), neutrophils (*r* = 0.270 [95% CI: 0.146–0.384]; *p* < 0.0001), NLR (*r* = 0.285 [95% CI: 0.163–0.399]; *p* < 0.0001), SII (*r* = 0.240 [95% CI: 0.116–0.357]; *p* = 0.0002), and LGI (*r* = 0.266 [95% CI: 0.143–0.381]; *p* < 0.0001) (Figure 3A–E).

### 3.6. Leukocyte Glucose Index Can Predict a MELD Score >9 in Patients with COVID-19

**Figure 4:**
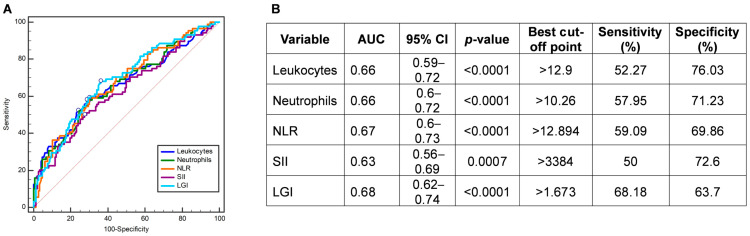
(**A**,**B**) Receiver operating characteristic (ROC) curves and accuracy evaluation of leukocytes, neutrophils, NLR, SII, and LGI in the predicting MELD score >9. Abbreviations: NLR, neutrophil-to-lymphocyte ratio; SII, systemic immune-inflammation index; MELD, Model of End-Stage Liver Disease Score; LGI, leukocyte glucose index.

Since MELD uses three target variables (bilirubin, creatinine, and INR), we set out to find out whether any of the previously analyzed inflammation-related parameters could predict MELD score values >9 in case any of the variables were missing for the calculation. ROC curves analysis showed that the AUC of LGI for predicting MELD score >9 was 0.68 (95% CI: 0.62–0.74; *p* < 0.0001; sensitivity = 68.18%; specificity = 63.7%) being higher than for the AUC of leukocytes (0.66), neutrophils (0.66), NLR (0.67), and SII (0.63) (Figure 4A,B). 

According to previous results of ROC curves, these inflammation-related parameters were selected to perform univariate regression analysis. The univariate regression showed that the Odds Ratios (OR) of leukocytes > 12.9, neutrophils > 10.26, NLR > 12.894, SII > 3384, and LGI > 1.673 were 3.47, 3.41, 3.34, 2.65, and 3.76 for predicting MELD score >9, respectively (Table 3). Nevertheless, only LGI > 1.673 was a predictor of MELD score >9 (OR = 2.42, 95% CI: 1.21–4.83; *P*_corrected_ = 0.045) for patients with COVID-19 in the multivariate logistic regression analysis (Table 3).

## 4. Discussion

Although a vaccine against COVID-19 is now available, SARS-CoV-2 outbreaks still occur today [20]. Hence, exploring biomarkers that identify the risk of COVID-19 mortality in different populations is essential. This study evaluated the predictive value of the MELD score for mortality in Mexican patients with COVID-19. The MELD score was significantly higher in the non-survivors than in survivors among Egyptian patients with chronic liver disease and COVID-19 [10]. A study in Turkey demonstrated that a MELD score of 18.5 predicts COVID-19 mortality with an AUC of 0.797, sensitivity = 99%, and specificity = 100% [9]. In contrast, our analysis of the MELD score in the Mexican population revealed that a cut-off point >9 had an AUC of 0.62 with a sensitivity of 53.68% and specificity of 78.42% for predicting COVID-19 mortality. The HR = 1.83 of MELD score for mortality in patients with SARS-CoV-2 infection obtained in our study was similar to the HR = 1.063 reported in a Turkish population [9]. Another study on MELD scores in COVID-19 patients in the United States reported that a MELD score ≥10 was associated with the 39% of mortality [8], while our study indicated that 53.7% of COVID-19 mortality was related to MELD score >9. The mortality rate of patients with COVID-19 in the United States, Turkey, and Mexico ranges from 3.14 to 61.51% [21]. The sensitivity (50%) and specificity (75%) of MELD score ≥10 for predicting SARS-CoV-2 mortality risk in the United States population [8] was very similar to the sensitivity (53.68%) and specificity (73.38%) of MELD score >9 observed in our analysis. Unlike these studies, our analysis included *P*_corrected_ values using Bonferroni corrections to compensate for the effect of multiple hypotheses testing. Interestingly, even when *p* values were adjusted, the MELD score consistently predicted COVID-19 mortality.

Although Kaplan–Meier survival curves showed that mortality was significantly associated with leukocytes, neutrophils, NLR, SII, MELD, and LGI according to their best cut-off points, the multivariate Cox regression analysis revealed that MELD score >9 was an independent predictor of COVID-19 mortality. Interestingly, we found a weak positive correlation between the values of MELD score and leukocytes, neutrophils, NLR, SII, and LGI in COVID-19 patients. The leukocytes, neutrophils, NLR, SII, and LGI were associated with clinical prognosis in SARS-CoV-2-infected subjects [16,17,22,23,24]. Nevertheless, these biomarkers were not predictors of mortality among the patients with SARS-CoV-2 infection despite a statistically significant AUC when the multivariate Cox regression analysis was performed. Other biomarkers of COVID-19, such as PLR [25], LDH/lymphocyte ratio [18], ALRI [11], ANRI [13], APRI [12], and BUN/creatinine [19], were analyzed in this study. Still, they showed no significant differences (*P*_corrected_ > 0.05) between survivors and non-survivors when the *p* values were adjusted. Therefore, these variables were excluded from the univariate Cox regression analysis. Some studies suggested that the association between biomarkers and COVID-19 depends heavily on population, study period, and quality of evidence [26,27,28]. 

The association between LGI and MELD score (OR = 2.42, 95% CI: 1.21–4.83; *P*_corrected_ = 0.045) suggested that, from an inflammatory point of view, LGI > 1.673 roughly corresponds to MELD score > 9 in patients with SARS-CoV-2 infection. Liver is an organ susceptible to SARS-CoV-2 infection [29]. The patients infected with SARS-CoV-2 included in this study had liver abnormalities because their AST and ALT levels were elevated, as previously reported [30]. These values showed no significant difference between surviving and non-surviving subjects. However, the patients had no liver injury because they did not show an increase in ALT or AST levels of at least three times the upper limit of normal, according to the definition suggested by Ding et al. [30]. Therefore, our study indicated that the MELD score could predict COVID-19 mortality even without liver injury in the patients. One hypothesis is that liver abnormalities or hepatic function secondary to inflammation during SARS-CoV-2 infection could be evaluated using the MELD score. This score has been shown to have a significant value in predicting clinical outcomes in the context of patients with human immunodeficiency virus (HIV) [31], hepatitis C virus [32], and hepatitis A virus [33] infections.

The present study has limitations: (1) it was retrospective, (2) single-center, and (3) had a relatively small sample size, possibly leading to biased results. Another limitation is that most of the studies on the biomarker for COVID-19 were conducted in European and Asian countries. Hence, the comparisons made in this study may needed to have been stronger. This study has some strengths, such as the ability of the MELD score to predict COVID-19 mortality compared with other biomarkers. Kaya et al. demonstrated that a high MELD score independently predicts the ICU admission and intubation requirements [9]. Another study could address the predictive value of the MELD score and the other biomarkers for the ICU admission or the need for respiratory support in the Mexican population. For the time being, our study included only patients admitted to the ICU with mechanical ventilation who met the other inclusion criteria.

## 5. Conclusions

In summary, this study suggested that MELD score >9 at admission can be an essential tool to help identify the mortality risk in patients infected with SARS-CoV-2 to early medical intervention. Incorporation and validation of the MELD score as a biomarker in the Mexican population can help standardize and propose optimal cut-off points worldwide.

## Figures and Tables

**Table 1:** Baseline characteristics.

Variable	Overall (*n* = 234)	Survival Group (*n* = 139)	Non-Survival Group (*n* = 95)	*p*-Value	Adjusted *p*-Value
Sex, female	71 (30.34%)	41 (29.49%)	30 (31.57%)	0.734	1
Age (years old)	63.27 ± 12.91	62.74 ± 12.678	65.23 ± 13.86	0.005	0.190
Height (m)	1.6 ± 0.9	1.6 ± 0.09	1.59 ± 0.1	0.910	1
Weight (kg)	73.25 (22.5)	74 (20.75)	67 (21.25)	0.220	1
BMI (kg/m^2^)	28.07 (6.1)	28.34 (5.57)	27.4 (7)	0.339	1
Diabetes	114 (48.71%)	79 (56.83%)	35 (36.84%)	0.003	0.114
Hypertension	112 (47.86%)	70 (50.35%)	42 (44.21%)	0.355	1
FiO_2_ (%)	50 (39)	41 (31)	60 (47)	0.008	0.304
SpO_2_ (%)	64 (39)	71 (38)	58.5 (35)	0.042	1
Hemoglobin (g/dL)	14.5 (2.7)	14.5 (2.4)	14.5 (3.2)	0.373	1
Hematocrit (%)	44 (8)	44 (7)	44 (7)	0.377	1
MCV	91 (6)	91 (6)	91 (5)	0.152	1
MCH	30.2 (2.3)	30.2 (2.3)	30.2 (2.2)	0.621	1
Leukocytes (×10^9^/L)	10.45 (7)	9.5 (6.7)	12.6 (7.3)	0.0001	**0.003**
Neutrophils (×10^9^/L)	8.74 (6.8)	7.72 (6.32)	11.39 (7.52)	0.0001	**0.003**
Lymphocytes (×10^9^/L)	0.81 (0.7)	0.86 (0.72)	0.76 (0.65)	0.122	1
Platelets (×10^9^/L)	252 (135)	246 (130)	267 (128)	0.355	1
INR	1.05 (0.17)	1.03 (0.16)	1.08 (0.18)	0.048	1
Glucose (mg/dL)	143 (120)	132.5 (105)	158 (142)	0.006	0.228
Urea (mg/dL)	45.8 (45.8)	38.2 (38.6)	57.1 (58.6)	0.008	0.304
Creatinine (mg/dL)	0.9 (0.7)	0.9 (0.5)	1.1 (1.1)	0.004	0.152
LDH (U/L)	850 (472)	820 (462)	916 (465)	0.008	0.304
BUN	21.4 (20.8)	17.75 (17.82)	27 (27.5)	0.002	0.076
TB (mg/dL)	0.6 (0.4)	0.5 (0.4)	0.6 (0.37)	0.275	1
DB (mg/dL)	0.3 (0.2)	0.2 (0.2)	0.3 (0.2)	0.004	0.152
IB (mg/dL)	0.3 (0.2)	0.3 (0.15)	0.3 (0.2)	0.186	1
AST (IU/L)	46 (44)	42.5 (47)	49 (40)	0.331	1
ALT (IU/L)	37 (38)	41.5 (45)	32 (30)	0.175	1
ALRI	60 (75.13)	53.31 (69.67)	65.04 (74.87)	0.241	1
APRI	0.48 (0.61)	0.49 (0.63)	0.46 (0.52)	0.785	1
ANRI	4.59 (7.07)	5.14 (8.18)	4.41 (5.85)	0.178	1
NLR	9.44 (12.15)	8.06 (11.02)	14.57 (15.19)	0.0001	**0.003**
PLR	284.67 (267.33)	265.83 (238.26)	329.78 (302.1)	0.058	1
SII	2525.03 (3503.23)	2149.52 (2759.38)	3017.84 (3976.29)	0.001	**0.038**
MELD	8 (5)	8 (3)	10 (7)	0.001	**0.038**
LGI	1.61 (1.61)	1.36 (1.4)	1.88 (2)	0.0001	**0.003**
LDH/LR	1038.27 (1091.26)	933.76 (955.81)	1193.15 (1237.85)	0.014	0.504
BUN/Cr	20.4 (12.79)	20.69 (13.19)	20 (14.63)	0.438	1

Data are mean ± standard deviation, median (interquartile range), *n* (%). *p* values were calculated by Student´s-*t* test, Mann–Whitney U test or Chi-squared test as appropriate. The bold values provide statistically significance (*p* < 0.05). Abbreviations: BMI, body mass index; FiO_2_, fraction of inspired oxygen; SpO_2_, oxygen saturation; MCV, mean corpuscular volume; MCH, mean corpuscular hemoglobin; INR, international normalized ratio; LDH, lactate dehydrogenase; BUN, blood urea nitrogen; TB, total bilirubin; DB, direct bilirubin; IB, indirect bilirubin; AST, Aspartate aminotransferase; ALT, Alanine aminotransferase ALRI, aspartate aminotransferase-to-lymphocyte ratio index; APRI, aspartate aminotransferase-to-platelet ratio index; ANRI, aspartate aminotransferase-to-neutrophil ratio index; NLR, neutrophil-to-lymphocyte ratio; PLR, platelets-to-lymphocyte ratio; SII, systemic immune-inflammation index; MELD, Model of End-Stage Liver Disease Score; LGI, leukocyte glucose index; LDH/LR, lactate dehydrogenase/lymphocyte ratio; BUN/Cr, BUN-to-creatinine ratio.

**Table 2:** Univariate and multivariate Cox regression analysis for predicting of COVID-19 mortality.

	Univariate				Multivariate			
Variable	HR	95% CI	*p*-Value	Adjusted *p*-Value	HR	95% CI	*p*-Value	Adjusted *p*-Value
Leukocytes	2.53	1.66–3.87	<0.0001	**0.0001**	1.55	0.62–3.86	0.342	1
Neutrophils	2.45	1.59–3.79	<0.0001	**0.0003**	0.96	0.36–2.51	0.939	1
NLR	2.46	1.58–3.82	<0.0001	**0.0003**	1.34	0.73–2.45	0.340	1
SII	2.49	1.52–4	0.0002	**0.001**	1.46	0.75–2.83	0.255	1
MELD	2.34	1.56–3.5	<0.0001	**0.0001**	1.83	1.2–2.8	0.005	**0.030**
LGI	2.17	1.45–3.25	0.0001	**0.0006**	1.2	0.74.1.95	0.444	1

Univariate Cox regression analysis was performed with the six variables that had statistically significant difference in the Table 1 using the multiple hypotheses (*P*_corrected_ < 0.05). Candidate predictors with statistically significant difference (*P*_corrected_ < 0.05) in univariate Cox regression analysis were included in multivariate Cox regression analysis. Hazard ratio (HR) and 95% Confidence Interval (CI 95%) were reported. The bold values provide statistically significance (*P*_corrected_ < 0.05). Abbreviations: NLR, neutrophil-to-lymphocyte ratio; SII, systemic immune-inflammation index; MELD, Model of End-Stage Liver Disease Score; LGI, leukocyte glucose index.

**Table 3:** Univariate and multivariate logistic regression analysis for predicting MELD score >9 in patients with COVID-19.

	Univariate				Multivariate			
Variable	OR	95% CI	*p*-Value	Adjusted *p*-Value	OR	95% CI	*p*-Value	Adjusted *p*-Value
Leukocytes	3.47	1.97–6.11	<0.0001	**0.0005**	1.64	0.49–5.46	0.415	1
Neutrophils	3.41	1.96–5.94	<0.0001	**0.0005**	0.89	0.25–3.11	0.864	1
NLR	3.34	1.92–5.82	<0.0001	**0.0005**	2.25	1.1–5.13	0.052	0.260
SII	2.65	1.52–4.6	0.001	**0.005**	0.98	0.43–2.21	0.965	1
LGI	3.76	2.14–6.59	<0.0001	**0.0002**	2.42	1.21–4.83	0.009	**0.045**

Univariate logistic regression analysis was performed with the five previously analyzed inflammation-related parameters that had statistically significant difference using the multiple hypotheses (*P*_corrected_ < 0.05). Candidate predictors with statistically significant difference (*P*_corrected_ < 0.05) in univariate Logistic regression analysis were included in multivariate logistic regression analysis. The Odds Ratio (OR) and 95% Confidence Interval (CI 95%) were reported. The bold values provide statistically significancy (*P*_corrected_ < 0.05). Abbreviations: NLR, neutrophil-to-lymphocyte ratio; SII, systemic immune-inflammation index; MELD, Model of End-Stage Liver Disease Score; LGI, leukocyte glucose index.

## Data Availability

Data supporting the reported results can be provided upon reasonable request by the corresponding author.
